# Causal relationship between hypothyroidism and peripheral neuropathy: a Mendelian randomization study of European ancestry

**DOI:** 10.3389/fendo.2024.1436823

**Published:** 2024-11-27

**Authors:** Xiping Duan, Tianchi Zhang, Ke Wang

**Affiliations:** ^1^ Acupuncture Anaesthesia Clinical Research Institute, Yueyang Hospital of Integrated Traditional Chinese and Western Medicine, Shanghai University of Traditional Chinese Medicine, Shanghai, China; ^2^ Laboratory of New Techniques of Restoration & Reconstruction, Institute of Traumatology & Orthopedics, Nanjing University of Chinese Medicine, Nanjing, Jiangsu, China

**Keywords:** hypothyroidism, peripheral neuropathy, carpal tunnel syndrome, polyneuropathies, Mendelian randomization, causal relationship

## Abstract

**Background:**

Metabolic disorders are significant risk factors for peripheral neuropathy (PN) diseases. However, current clinical observational studies cannot fully determine the causal relationships between hypothyroidism (HT) and PN diseases.

**Methods:**

We performed univariate Mendelian randomization (MR) analyses using single nucleotide polymorphisms (SNPs) associated with hypothyroidism and two diseases clinically presented as HT (autoimmune thyroid disease and benign neoplasm of the pituitary gland and craniopharyngeal duct) as instrumental variables. We selected eight peripheral neuropathy diseases (diabetic neuropathy, nerve root/plexus disorder, carpal tunnel syndrome, polyneuropathies, sciatica with lumbago, trigeminal neuralgia, postherpetic neuralgia, small fiber neuropathy) as outcomes. Genetic data were sourced from authoritative genome-wide association study (GWAS) datasets. We primarily used the inverse variance-weighted (IVW) method and conducted a comprehensive sensitivity analysis to ensure robustness.

**Results:**

The IVW results indicated that HT was significantly associated with an increased risk of diabetic peripheral neuropathy (OR = 1.22, *p* = 6.49E-05). HT was also significantly linked to nerve root/plexus disorder (OR = 1.04, p = 6.43E-06) and carpal tunnel syndrome (OR = 1.04, *p* = 0.004), but appeared to be a potential protective factor for polyneuropathies (OR = 0.93, *p* = 0.0009). Additionally, autoimmune thyroid disease (AITD) was identified as a potential risk factor for carpal tunnel syndrome (OR = 13.79, *p* = 0.006) and a protective factor for polyneuropathies (OR = 0.0011; *p* = 4.44E-5).

**Conclusions:**

This study provides genetic evidence supporting potential causal links between hypothyroidism and various peripheral neuropathy diseases.

## 1 Introduction

Hypothyroidism (HT) can be idiopathic or caused by conditions such as autoimmune thyroiditis, pituitary neoplasms, dysfunction, or secondary to thyroid disorder treatments (1). In Europe, the lifetime risk of overt hypothyroidism is around 5%, often following subclinical hypothyroidism, which affects up to 9% of the population (2, 3). It is well-documented that metabolic disorders are a major cause of peripheral neuropathy (PN) (4), and HT may initially manifest as neuropathy, especially in autoimmune cases (5). Studies have consistently shown a strong link between HT and neurological complications (6–8). The prevalence of peripheral nerve disorders in HT patients ranges from 10% to 70%, with symptoms varying from mononeuropathy to polyneuropathy, often accompanied by neuropathic pain (9–11). Additionally, some HT patients experience painful extremities indicative of small-fiber neuropathy (12). However, the specific phenotype of PN and neuropathic pain related to HT is still debated. Subclinical hypothyroidism (SCH) is defined by a TSH level above the normal upper limit with a normal free thyroxine (FT4) level. SCH is prevalent among patients with diabetic peripheral neuropathy (DPN) (6, 13). In European clinical observations, HT patients often show no obvious neuromuscular deficits in routine tests (14). Research by Nebuchennykh et al. indicates that HT may affect various nerve fiber types; many patients exhibit both large and small fiber involvement, while others have only small fiber polyneuropathy (15). A cross-sectional study in Mexico found a link between HT and headache disorders, including occipital and trigeminal neuralgia (16). Conversely, a Brazilian case-control study identified HT as a predictor for non-neuropathic orofacial conditions rather than neuralgia (17). Similarly, another study found that HT was associated with lower odds of developing neuropathic pain (18).

Controlling for confounding factors is a significant challenge in real-world clinical studies examining hormone therapy (HT)-induced peripheral neuropathy (PN) diseases. Conventional observational studies may have their validity undermined by these factors and reverse causation bias (19). Mendelian randomization (MR) not only addresses the limitations of observational studies by simulating a randomized controlled trial, but also offers insights beyond those studies. Therefore, assessing the causal relationship between HT and PN diseases using the MR method is crucial. In this study, we conducted two-sample MR analyses to explore the direct causal relationship between hypothyroidism and PN diseases.

## 2 Materials and methods

### 2.1 Study design

**Figure 1:**
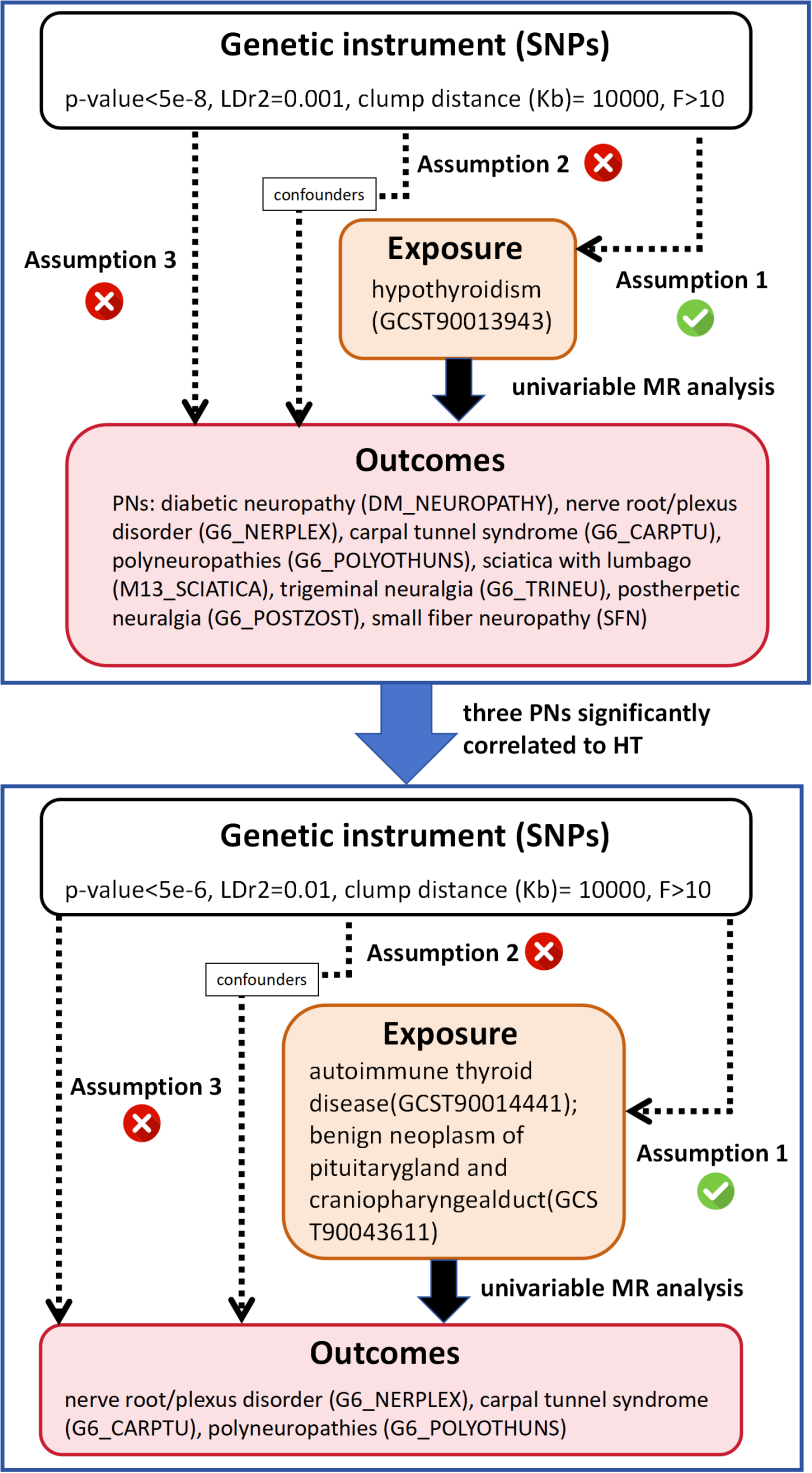
Study design overview.

This study was structured into two phases as shown in **Figure 1**. Initially, we employed two-sample univariate MR to analyze the causality between HT and eight different PN diseases. Based on the initial MR results, we identified PN diseases significantly associated with HT and further investigated their causal relationships with two diseases clinically presenting as HT (autoimmune thyroid disease and benign neoplasm of the pituitary gland and craniopharyngeal duct). The MR analysis was grounded in three fundamental assumptions: 1) the chosen single nucleotide polymorphisms (SNPs) must show a statistically significant correlation with the exposure; 2) the SNPs should be independent of any confounders related to both the exposure and the outcome; 3) the SNPs should influence the outcome solely through the exposure and not through other direct or indirect pathways. Our procedures adhered strictly to the guidelines of the Strengthening the Reporting of Observational Studies in Epidemiology Using Mendelian Randomization (STROBE-MR) (20).

### 2.2 Data sources

We obtained summary Genome-Wide Association Study (GWAS) statistics from publicly accessible databases, detailed in **Table 1**. The summary data for hypothyroidism came from a cohort of 405,357 individuals of British ancestry as a train set (21). We used hypothyroidism (congenital or acquired) from FinnGen R5 as the exposure for the validation group. Because hypothyroidism often results from autoimmune thyroid disease or benign neoplasm of the pituitary gland, we also selected summary-level data for associated SNPs of these two diseases. The summary statistics for autoimmune thyroid disease came from a cohort of 607 cases and 324,074 controls of European (U.K.) ancestry (22). The statistics for benign neoplasm of the pituitary gland were derived from a cohort of 285 cases and 456,063 controls of European (U.K.) ancestry (23). All data were sourced exclusively from the GWAS Catalog (https://www.ebi.ac.uk/gwas/), IEU open GWAS catalog (https://gwas.mrcieu.ac.uk/) and FinnGen (https://www.finngen.fi/en/access_results). British ancestry data were extracted from UK Biobank dataset (http://www.ukbiobank.ac.uk), a prospective cohort study with deep genetic and phenotypic data collected on approximately 500,000 individuals from across the United Kingdom (24). FinnGen is a large public-private partnership aiming to collect and analyze genome and health data from 500,000 Finnish biobank participants.

**Table 1:** information of datasets in study.

Phenotype of exposure /outcome	GWAS ID	Sample size (cases/controls)	Ethnicity	Year of publication	First author	PMID
Hypothyroidism or myxoedema	GCST90013943	405357	U.K.	2021	Mbatchou J	34017140
Hypothyroidism (congenital or acquired)	finn-b-HYPOTHYROIDISM	26342+59827	Finland	2021	NA	NA
Autoimmune thyroid disease	GCST90014441	607+324074	U.K.	2021	Glanville KP	34278373
Benign neoplasm of pituitary gland and craniopharyngeal duct	GCST90043611	285+456063	U.K.	2021	Jiang L	34737426
Diabetic neuropathy	DM_NEUROPATHY	1769+190836	Finland	2022	NA	NA
Nerve root/plexus disorder	G6_NERPLEX	51643+360538	Finland	2023	NA	NA
Nerve, nerve root and plexus disorders	ukb-d-G6_NERPLEX	10898+350296	U.K.	2018	Neale lab	NA
Carpal tunnel syndrome	G6_CARPTU	24766+360538	Finland	2023	NA	NA
Carpal tunnel surgery	ukb-b-17788	4858+458075	U.K.	2018	Ben Elsworth	NA
Polyneuropathies	G6_POLYOTHUNS	6027+405136	Finland	2023	NA	NA
Sciatica with lumbago	M13_SCIATICA	20699+294770	Finland	2023	NA	NA
Trigeminal neuralgia	G6_TRINEU	1777+360538	Finland	2023	NA	NA
Postherpetic neuralgia	G6_POSTZOST	356+360538	Finland	2023	NA	NA
Small fiber neuropathy	SFN	683+405136	Finland	2023	NA	NA

NA, Not available.

For the eight PN diseases, we extracted data for nerve root/plexus disorder (G6_NERPLEX) with 51643 cases and 360538 controls, carpal tunnel syndrome (G6_CARPTU) with 24766 cases and 360538 controls, polyneuropathies (G6_POLYOTHUNS) with 6027 cases and 405136 controls, sciatica with lumbago (G6_SCIATICA) 6027 cases and 405136 controls, trigeminal neuralgia (G6_TRINEU) 1777 cases and 360538 controls, postzoster neuralgia (G6_POSTZOST) 356 cases and 360538 controls, and small fibre neuropathy (SFN) with 683 cases and 405136 controls from FinnGen R10. Diabetic neuropathy (DM_NEUROPATHY) with 1769 cases and 190836 controls were sourced from FinnGen R6 (25). The above data are all from the analysis of FinnGen biobank dataset (https://www.finngen.fi/en), and these FinnGen data were downloaded from online website (https://www.finngen.fi/en/access_results). For outcomes in validation group, we reviewed nerve, nerve root, and plexus disorders (ukb-d-G6_NERPLEX) with 51643 cases and 360538 controls and carpal tunnel surgery (ukb-b-17788) with 4858 cases and 458057 controls from MRC-IEU. These eight PN diseases were defined based on the International Classification of Diseases (ICD) codes (ICD-10) (**Supplementary Table 1** in the **Supplementary Materials**).

### 2.3 Genetic instrument selection

Before conducting MR analyses, we selected SNPs strongly associated with hypothyroidism and its related conditions as instrumental variables (IVs) respectively, using a significance threshold of *p*<5E-8. If only a few SNPs met this criterion, a higher cutoff of *p*<5E-6 was applied. To ensure independence from linkage disequilibrium (LD), we set an LD metric of r² = 0.01 and a clumping distance of 1000 kb, removing any palindromic SNPs with intermediate allele frequencies. We then utilized the LDtrait Tool website (https://ldlink.nih.gov/?tab=ldtrait) to identify SNPs associated with potential confounders—such as smoking, alcohol consumption, and BMI—and manually excluded them. The strength of the selected IVs was measured using an F-statistic (beta²/se²), considering only those with an F-statistic > 10 as valid and reliable.

### 2.4 Statistical analysis

We initially treated hypothyroidism as an exposure factor and explored its causal relationship with several PN diseases. Additionally, we examined the causal links between two diseases presenting as HT and their associated PN diseases. To identify and remove outliers, Radial MR was performed prior to MR analysis (26). For univariate MR, we employed random effects inverse variance weighted (IVW) regression—considered the most efficient method under complete IV assumptions (27)—as our primary analysis tool. Given the presence of heterogeneity, a random effects model was used. Four supplementary sensitivity analyses—MR-Egger regression, weighted median, weighted mode, and simple mode—were conducted to confirm the robustness of the causality results across different contexts. To ensure the directionality accuracy of statistically significant results, we incorporated the Steiger directionality test. To minimize false positives, p-values were adjusted using the Bonferroni test. In the validation group, the Benjamini-Hochberg (BH) method was used to calculate the false discovery rate (FDR), considering the adjusted p-value as the conclusive result. All results were expressed as odds ratios (OR) with 95% confidence intervals (CIs) and adjusted P-values.

For sensitivity analysis, we employed MR-Egger regression method and the leave-one-out approach. The P-value from the MR-Egger intercept test was used to evaluate potential horizontal pleiotropy in the IVs (28). Additionally, Cochrane’s Q statistic was utilized to assess the consistency of SNP estimates across each MR association. We also conducted a weighted median test to verify the directionality of each significant univariate MR result. All analyses were conducted using the TwoSample MR (29) and RadialMR (26) packages in R software (version 4.3.1).

## 3 Results

### 3.1 Instrumental variables selection

We selected SNPs based on P-value screening and LDtrait Tool filtering, ensuring a strong association with the exposure and no significant correlation among them. SNPs related to smoking, alcohol consumption, and BMI were excluded after evaluation using the LDtrait Tool. Overall, we identified 128 SNPs associated with hypothyroidism or myxoedema (GCST90013943) and hypothyroidism (congenital or acquired) (finn-b-HYPOTHYROIDISM), 17 SNPs linked to autoimmune thyroid disease (GCST90014441) and 14 SNPs related to benign neoplasm of pituitary gland and craniopharyngeal duct (GCST90043611). Among these IVs, no weak instrument bias existed (F-statistics > 10). Before conducting univariate MR analysis, RadialMR was used to remove potential outliers. Comprehensive details of all selected SNPs are available in **Supplementary Table 1**.

### 3.2 Univariable MR for the causal relationship between HT and PN diseases

**Figure 2:**
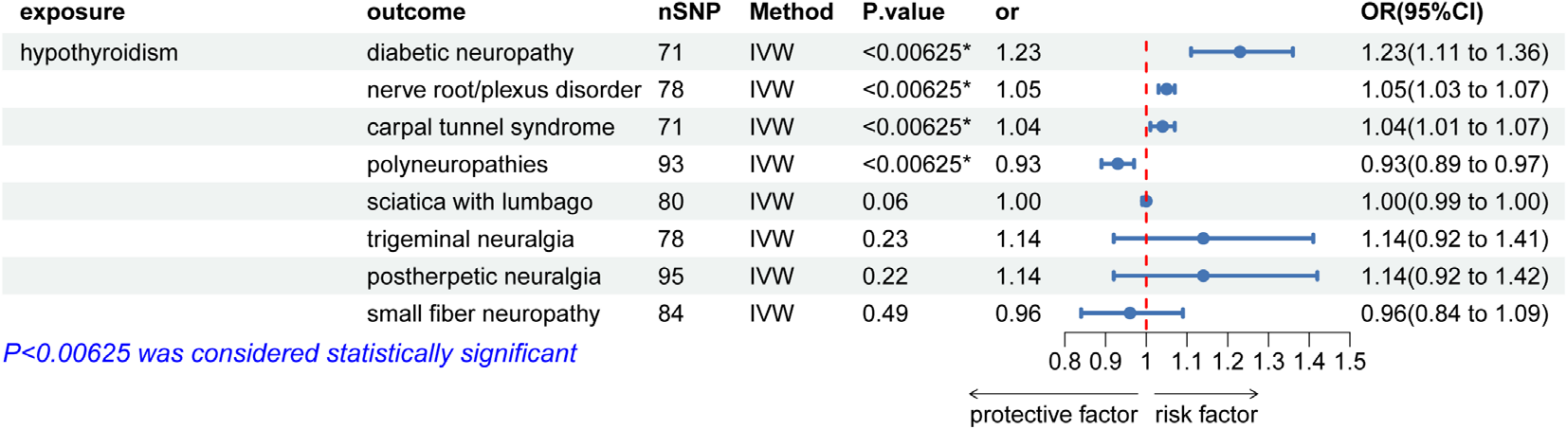
Causal effects for hypothyroidism on peripheral neuropathy. IVW, inverse-variance weighted; *IVW P-value (adjusted) ≤ 0.00625; &, IVW P-value (adjusted) ≤ 0.0125.

As illustrated in **Figure 2**, the univariable MR analysis revealed causal relationships between HT and eight PN diseases, with results from other four MR methods aligning consistently with IVW (**Table 2**; **Supplementary Figure 4**). With eight outcomes, the Bonferroni test was applied, adjusting the P-value to 0.00625 (0.05/8). The IVW results provide substantial genetic evidence that HT is significantly associated with an increased likelihood of diabetic polyneuropathy (DPN) (IVW: OR = 1.22, 95% CI: 1.11 – 1.35; *p* = 6.49E-5). Additionally, HT is genetically linked to an increased risk of nerve root/plexus disorders (IVW: OR = 1.04, 95% CI: 1.02 – 1.07; *p* = 6.43E-6) and carpal tunnel syndrome (IVW: OR = 1.04, 95% CI: 1.01 – 1.07; *p* = 0.004), and potentially acts as a protective factor against polyneuropathies (IVW: OR = 0.93, 95% CI: 0.89 – 0.97; *p* = 0.0009). No causal relationships were found in other PN diseases.

**Table 2:** The genetic causal association between HT and PNs.

Exposure	Outcome	Analysis method	Odds ratio	OR 95%CI	P value
Hypothyroidism or myxoedema	Diabetic neuropathy*	Inverse variance weighted	1.23	1.11~1.36	6.50E-5
		MR Egger	1.24	0.93~1.64	0.15
		Weighted median	1.23	1.06~1.45	5.84E-3
		Simple model	1.35	0.92~2.01	0.13
		Weighted model	1.05	0.79~1.39	0.76
	Nerve root/plexus disorder*	Inverse variance weighted	1.05	1.03~1.07	6.43E-6
		MR Egger	1.04	0.98~1.11	0.18
		Weighted median	1.04	1.01~1.07	2.56E-3
		Simple model	1.05	0.97~1.12	0.21
		Weighted model	1.03	0.98~1.09	0.24
	Carpal tunnel syndrome*	Inverse variance weighted	1.04	1.01~1.07	4.57E-3
		MR Egger	1.06	0.97~1.16	0.22
		Weighted median	1.07	1.02~1.11	2.24E-3
		Simple model	1.08	0.98~1.19	0.12
		Weighted model	1.08	1.01~1.15	0.03
	Polyneuropathies*	Inverse variance weighted	0.93	0.89~0.97	9.00E-4
		MR Egger	0.93	0.84~1.02	0.12
		Weighted median	0.90	0.83~0.96	3.02E-3
		Simple model	0.92	0.78~1.09	0.35
		Weighted model	0.88	0.79~0.99	0.03
	Sciatica with lumbago	Inverse variance weighted	0.99	0.99~1.00	0.06
		MR Egger	0.99	0.99~1.00	0.62
		Weighted median	1.00	0.99~1.00	0.75
		Simple model	1.00	0.99~1.00	0.29
		Weighted model	1.00	0.99~1.00	0.50
	Trigeminal neuralgia	Inverse variance weighted	1.13	0.92~1.41	0.23
		MR Egger	1.20	0.67~2.17	0.53
		Weighted median	1.20	0.84~1.71	0.31
		Simple model	1.15	0.54~2.44	0.71
		Weighted model	1.35	0.81~2.26	0.25
	Postherpetic neuralgia	Inverse variance weighted	1.14	0.92~1.41	0.22
		MR Egger	1.20	0.67~2.16	0.55
		Weighted median	1.21	0.86~1.70	0.27
		Simple model	1.31	0.63~2.73	0.47
		Weighted model	1.42	0.88~2.31	0.16
	Small fiber neuropathy	Inverse variance weighted	0.96	0.84~1.09	0.49
		MR Egger	1.06	0.80~1.42	0.68
		Weighted median	1.01	0.81~1.25	0.93
		Simple model	1.06	0.69~1.63	0.79
		Weighted model	1.03	0.81~1.30	0.83

* According to the method of inverse variance weighted, the association between exposure and outcome is statistically significant (P < 0.05).

**Figure 3:**
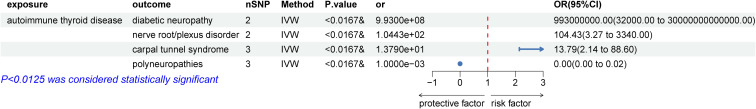
Plots of leave-one-out analyses of a genetically causal association between HT and PNs.

**Figure 4:**
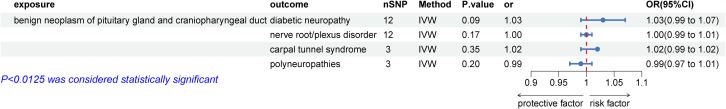
Causal effects for benign neoplasm of the pituitary gland and craniopharyngeal duct on peripheral neuropathy genetically associated with hypothyroidism. IVW, inverse-variance weighted.

We then examined the causal relationships between autoimmune thyroid disease (AITD) and peripheral neuropathy disorders that showed statistically significant associations with HT. The P-value was adjusted to 0.0125 (0.05/4) using the Bonferroni test. The results, displayed in **Figure 3**, were similar to the initial study. Due to heterogeneity, a random effects model was employed to analyze the causal relationships between AITD and carpal tunnel syndrome (CTS). The results showed that AITD performed as a potential risk factor for DPN (OR = 9.93E+8, 95% CI: 3.2E+5 – 3E+13; *p* = 88.28E-5), CTS (OR = 13.79, 95% CI: 2.14 – 88.63; *p* = 0.006), and a potential protective factor for polyneuropathies (IVW: OR = 1E-3, 95% CI: 6.68E-5 – 0.02; *p* = 4.44E-5), but no causal relationship between AITD and nerve root/plexus disorder (IVW: OR = 0.09, 95% CI: 0.004 – 2.47; *p* = 0.16). However, as shown in **Figure 4**, no causal relationships were found between HT and benign neoplasm of the pituitary gland or craniopharyngeal duct, DPN (OR = 1.03, 95% CI: 1.00 –1.07; *p* = 0.09), CTS (OR = 1.01, 95% CI: 0.99 – 1.02; *p* = 0.35), nerve root/plexus disorder (IVW: OR = 1.01, 95% CI: 1.00 – 1.01; *p* = 0.17), polyneuropathies (IVW: OR = 0.99, 95% CI: 0.97 – 1.01; *p* = 0.20).

To strengthen our findings, we conducted a validation analysis between HT and eight PN diseases. Due to limited data on outcomes across different ancestry, we only performed the validation for HT with nerve root/plexus disorder (IVW: OR = 1.001, 95% CI: 1.000 – 1.001; *p* = 0.34) and carpal tunnel syndrome (CTS) (IVW: OR = 1.001, 95% CI: 1.000 – 1.002; *p* = 0.08). Although the results were not statistically significant, the ORs showed consistency with our main results using IVW, MR-Egger, and Weighted median methods.

**Figure 5:**
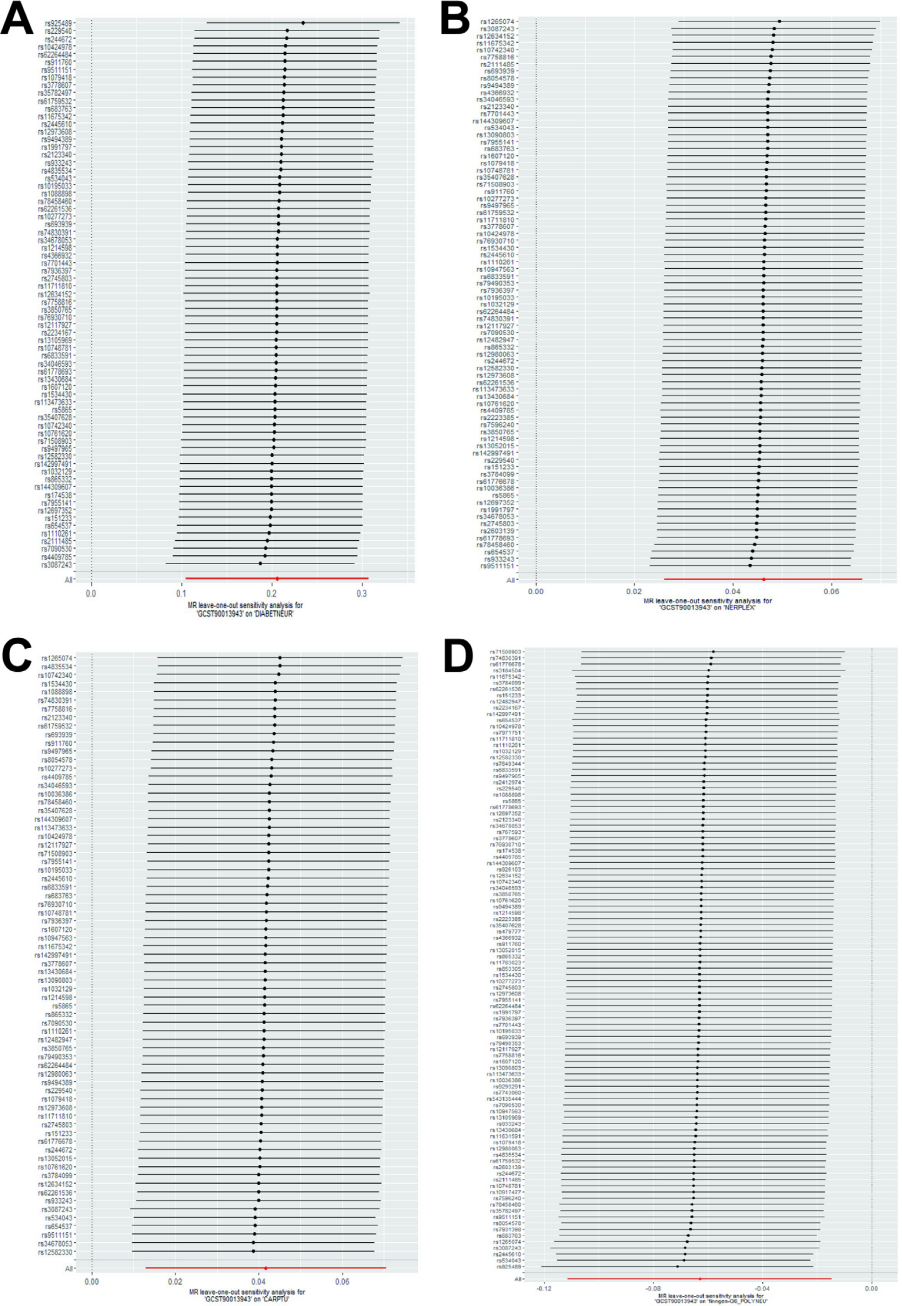
Plots of leave-one-out analyses of a genetically causal association between hypothyroidism and peripheral neuropathy. The error bars indicate the 95% confidence interval. **(A)** hypothyroidism on diabetic neuropathy; **(B)** hypothyroidism on nerve root/plexus disorder; **(C)** hypothyroidism on carpal tunnel syndrome; **(D)** hypothyroidism on polyneuropathies.

We performed the Steiger directionality test after the univariable MR analysis, confirming that all results were in the correct positive direction (**Table 3**). Additionally, a series of sensitivity analyses assessed the robustness of these findings. Heterogeneity was detected in the analysis between autoimmune thyroid disease (AITD) and nerve root/plexus disorder, and CTS, according to Cochran’s Q statistic, but no horizontal pleiotropy was detected as per the MR-Egger intercept test (**Table 4**). The funnel plots indicated a symmetric distribution (**Supplementary Figure 4**), and the “leave-one-out” method showed that removing any SNP had minimal impact on the overall error line (**Figure 5**; **Supplementary Figures 1**, **2**).

**Table 3:** Steiger directionality test results.

Exposure	Outcome	SNP r2.exposure	SNP r2.outcome	Steiger p.value	Correct causal direction
hypothyroidism or myxoedema	diabetic neuropathy	0.01217	0.00043	9.86e-232	TRUE
hypothyroidism or myxoedema	nerve root/plexus disorder	0.01189	0.00019	0	TRUE
hypothyroidism or myxoedema	carpal tunnel syndrome	0.01141	0.00015	0	TRUE
hypothyroidism or myxoedema	polyneuropathies	0.07727	0.00020	0	TRUE
autoimmune thyroid disease	diabetic neuropathy	0.00588	0.00031	0.023	TRUE
autoimmune thyroid disease	carpal tunnel syndrome	0.00048	0.00015	2.16e-5	TRUE
autoimmune thyroid disease	polyneuropathies	0.00057	0.00005	8.76e-13	TRUE

**Table 4:** Sensitivity analysis of the causal association between HT and PNs.

Exposure	Outcome	Cochran Q test		MR-Egger pval
		Q	Q-pval	
Hypothyroidism or myxoedema	Diabetic neuropathy	67.52	0.56	0.97
Hypothyroidism or myxoedema	Nerve root/plexus disorder	60.21	0.92	0.94
Hypothyroidism or myxoedema	Carpal tunnel syndrome	50.83	0.96	0.74
Hypothyroidism or myxoedema	Polyneuropathies	73.62	0.92	0.97
Hypothyroidism or myxoedema	Sciatica with lumbago	68.56	0.87	0.16
Hypothyroidism or myxoedema	Trigeminal neuralgia	70.78	0.73	0.83
Hypothyroidism or myxoedema	Postherpetic neuralgia	70.63	0.68	0.87
Hypothyroidism or myxoedema	Small fiber neuropathy	94.28	0.47	0.42
Autoimmune thyroid disease	Nerve root/plexus disorder	18.16	<0.01	0.15
Autoimmune thyroid disease	Carpal tunnel syndrome	6.12	0.03	0.24
Autoimmune thyroid disease	Polyneuropathies	0.31	0.86	0.99
Benign neoplasm of pituitary gland and craniopharyngeal duct	Nerve root/plexus disorder	6.02	0.87	0.25
Benign neoplasm of pituitary gland and craniopharyngeal duct	Carpal tunnel syndrome	13.72	0.25	0.10
Benign neoplasm of pituitary gland and craniopharyngeal duct	Polyneuropathies	4.03	0.97	0.34

## 4 Discussion

In our MR study of hypothyroidism and eight PN diseases, three MR analyses were conducted by using European ancestry GWAS data to detect the relationship between hypothyroidism and PN diseases risk. we found that hypothyroidism is a risk factor of diabetic neuropathy, nerve root/plexus disorder, and carpal tunnel syndrome, while acted as a oppose causality for polyneuropathy. Further analysis showed that autoimmune thyroid disease is a risk factor of carpal tunnel syndrome. However, we observed no statistically significant association between benign neoplasm of the pituitary gland and craniopharyngeal duct and PN diseases.

Thyroid hormones play a significant role in the normal functioning of the nervous system, and abnormalities in thyroid hormone levels can be associated with various neuropathies (30). Causes of hypothyroidism include primary, central, and extra-thyroidal hypothyroidism (1). Clinical observational studies suggest an association between hypothyroidism and peripheral neuropathy (8, 9, 11–13). The underlying mechanisms through which hypothyroidism contributes to neuropathy remain insufficiently explored. Recent research has identified that impaired transport of thyroid hormones to the brain results in cerebral hypothyroidism, which subsequently induces astrogliosis and disrupts both the metabolic function and mitochondrial respiration of astrocytes (31). One proposed mechanism for nerve damage associated with hypothyroidism involves fluid retention, which may lead to tissue swelling and the subsequent compression of peripheral nerves (32). The reduced transport of the slow component ‘a’ in hypothyroidism could explain peripheral neuropathy with axonal degeneration, often seen in severe myxedema (33). Research in murine demyelination models indicates that thyromimetics might protect against oligodendrocyte death, demyelination, axonal degeneration, and stimulate remyelination in multiple sclerosis (34). In Hashimoto’s thyroiditis, often presenting as hypothyroidism in children, severe and prolonged hypothyroidism may lead to bilateral peripheral polyneuropathy (35). Regarding diabetic neuropathy, carpal tunnel syndrome, and polyneuropathies, earlier clinical observations align with our findings. Patients with subclinical hypothyroidism are more likely to develop severe diabetic peripheral neuropathy (DPN), and their HbA1c levels are higher compared to those without SCH (13). Moreover, subclinical hypothyroidism patients are often diagnosed with hypertension (36). Clinical studies also indicate that hypertension, HbA1c levels, and TSH levels are risk factors for DPN (37). Furthermore, diabetes is a common cause of polyneuropathies, and studies suggest an association between subclinical hypothyroidism and end-stage diabetic polyneuropathy in type 2 diabetes patients, although the severity of neuropathy is not significantly correlated with TSH levels (38). A medical history of hypothyroidism indicates a risk for developing peripheral polyneuropathies (39), manifested as moderate myopathy and sensorimotor neuropathy. A case study of a 30-year-old male with severe hypothyroidism due to chronic autoimmune thyroiditis revealed severe sensorimotor polyneuropathy with both axonal and demyelinating features through nerve conduction studies (10). Carpal tunnel syndrome (CTS) is a common entrapment neuropathy caused by compression of the median nerve at the wrist. Risk factors for CTS include female sex, diabetes, hypothyroidism, obesity, arthritis, hemodialysis, acromegaly, and pregnancy (40, 41). While hypothyroidism is often considered a cause and comorbidity of CTS (42, 43), one meta-analysis found only a modest association after adjusting for potential confounders (44). Similarly, an 11-year study in a South Korean population found that hypothyroidism was not a risk factor for CTS (45).

There are relatively few reports on the correlation between hypothyroidism and nerve root/plexus disorder, sciatica with lumbago, trigeminal neuralgia, postherpetic neuralgia, and SFN. A prospective cohort study from China found that a correlation between lower back pain (LBP) with physical inactivity and hypothyroidism in pregnant women, and suggested that increase physical activity and normalize thyroid function and weight gain during pregnancy can have beneficial effects on LBP (46). A case report reported a hypothyroidism patient with non-specific LBP as an initial clinical manifestation (47). Nerve root/plexus disorders are typically caused by pressure on the nerves entering and exiting the spinal cord; however, there is a lack of literature exploring the link between hypothyroidism and these disorders. Only one clinical case study observed that hypothyroid patients had higher odds of developing complications with bad lumbar intervertebral disc morphology (48). Although a genetic causal relationship between hypothyroidism and nerve root/plexus disorders was identified, further clinical evidence is needed. Our results indicate no relationship between hypothyroidism and sciatica with lumbago. Nevertheless, hypothyroidism is strongly associated with higher cumulative Pfirrmann grades, a system for assessing lumbar degenerative disc disease (49). Our study suggests no correlation between hypothyroidism and two types of neuralgia; however, one study in a Mexican population noted a prevalence of hypothyroidism in occipital and trigeminal neuralgia (16). Conversely, another study involving 1,565 patients with extremity amputations found that hypothyroidism was associated with lower odds of developing neuropathic pain (18). Research on hypothyroidism and neuralgia is limited and should be expanded. In this study, no association was observed between hypothyroidism and SFN. However, four small sample size and the single centric studies suggested that patients with hypothyroidism or autoimmune thyroiditis tend to have small fiber nerve dysfunction or SFN (12, 50–52). Inconsistent results may be due to the following reasons. First, MR analysis relies on genetic variants as instrumental variables. It is possible that the genetic variants shared between hypothyroidism and SFN are not strongly associated with both conditions, leading to insufficient power to detect a causal effect. Second, SFN has a multitude of risk factors and these confounding factors may have influenced the results of this study during the analysis. Third, hypothyroidism may influence the occurrence of SFN through non-genetic pathways, such as by affecting metabolism, immune responses, or other physiological processes, which might not be fully captured in MR studies.

### 4.1 Strengths and limitations

This study employed a two-sample Mendelian Randomization (MR) design to investigate the genetic association between hypothyroidism and peripheral neuropathy diseases. This research presents several key findings. To our knowledge, this is the first MR study to demonstrate a genetic causal relationship between hypothyroidism and peripheral neuropathy diseases. We further explored the genetic association between hypothyroidism and three related peripheral neuropathy diseases, aiming to provide evidence-based guidelines for clinical practice and enhance our biological understanding of the underlying mechanisms. Additionally, the genome-wide association study (GWAS) data utilized in this study were sourced from a reputable consortium, providing robust statistical power to accurately estimate association. We applied stringent criteria for significance using the Bonferroni test, conducted sensitivity analyses, and assessed the strength of instrumental variables, ensuring the reliability of our results.

However, the study has several limitations. Firstly, the GWAS data were solely from individuals of European descent (U.K. and Finland), potentially limiting the generalizability of our findings. Secondly, the heterogeneity observed in the association between autoimmune thyroid disease (AITD) and nerve root/plexus disorder, as well as CTS, was indicated by Cochran’s Q test. MR Egger regression did not detect any significant pleiotropy, suggesting that the genetic instruments were primarily associated with the exposure rather than directly with the outcomes. Furthermore, the leave-one-out analysis yielded stable results, indicating that the overall effect estimate was not driven by any single genetic variant. Given that MR analyses presuppose linearity, potential nonlinear associations—such as those related to age, body status, sex, or genetic variants originating from different populations, experimental setups, and analytical platforms—may contribute to heterogeneity in this study. Lastly, we excluded single nucleotide polymorphisms (SNPs) associated with known confounders, but further studies are required to address other unknown confounders.

### 4.2 Conclusions

Using extensive genetic summary data, we conclude that hypothyroidism is strongly associated with risk factors for diabetic neuropathy, nerve root/plexus disorder, and carpal tunnel syndrome, whereas it appears to be a protective factor against polyneuropathies. Autoimmune thyroid disease is strongly linked to a risk of carpal tunnel syndrome but seems to be protective against polyneuropathies. Nonetheless, we found no statistically significant association between benign neoplasm of the pituitary gland and craniopharyngeal duct and peripheral neuropathy diseases.

## Data Availability

Publicly available datasets were analyzed in this study. This data can be found here: https://www.ebi.ac.uk/gwas/ and https://www.finngen.fi/en/access_results.
