# Supplementary material for: Causal relationship between hypothyroidism and peripheral neuropathy: a Mendelian randomization study of European ancestry
Source: Front Endocrinol (Lausanne). 2024 Nov 27;15:1436823. doi: 10.3389/fendo.2024.1436823 (PMC11631618; doi:10.3389/fendo.2024.1436823)
Supplement: Supplementary file 1 [file DataSheet1.docx]

Supplementary Material

# 1 **Supplementary Table**

## **Supplementary Table 1 Details of definitions for neurological disorders**

| neurological disease | GWAS ID | ICD codes (ICD-10) | Sample size  (cases/controls) | definition |
| --- | --- | --- | --- | --- |
| diabetic neuropathy | DM_NEUROPATHY | E1[0-4]4 | 1769+190836 | a chronic, pathological complication associated with diabetes mellitus, where nerve damages are incurred due to diabetic microvascular injury involving small blood vessels that supply these nerves, resulting in peripheral and/or autonomic nerve dysfunction |
| nerve root/plexus disorder | G6_NERPLEX | G50-G59, G53.0*B02.20, G53.0*B02.21 | 51643+360538 | peripheral nervous system disease: A disease involving the peripheral nervous system, including nerve roots and plexus, peripheral nerve entrapment syndrome, nerve root compression |
| nerve, nerve root and plexus disorders | ukb-d-G6_NERPLEX | G54.9 | 10898+350296 |  |
| carpal tunnel syndrome | G6_CARPTU | G56.0 | 24766+360538 | entrapment of the median nerve in the wrist that is characterized by numbness, tingling and painful movement |
| carpal tunnel surgery | ukb-b-17788 |  | 4858+458057 |  |
| polyneuropathies | G6_POLYOTHUNS | G60-G64 | 6027+405136 | Including hereditary and idiopathic neuropathies, inflammatory polyneuropathy, other polyneuropathy, polyneuropathy caused by diseases classified elsewhere, other disorders of the peripheral nervous system. |
| sciatica with lumbago | M13_SCIATICA | M54.3, M54.4 | 20699+294770 | a syndrome characterized by pain in the sciatic nerve pathway and distribution area |
| trigeminal neuralgia | G6_TRINEU | G50.0 | 1777+360538 | a syndrome refers to the facial pain distribution of more than 1 branch of the trigeminal nerve which characterized by paroxysmal sharp, knife-like severe pain that can last up to 2 minutes, and/or persistent facial pain without associated neurological deficits |
| Postherpetic neuralgia | G6_POSTZOST | G53.0, G53.0*B02.20, G53.0*B02.21 | 356+360538 | cranial nerve palsy: Injury to any of the cranial nerves or their nuclei in the brain resulting in muscle weakness. |
| Small fiber neuropathy | SFN | G60.8, G62.8, G63.3 | 683+405136 | Including Reggie's syndrome, Hereditary sensory nerve,Nelaton syndrome,Morvan disease, Giant axonal neuropathy, Sensory peripheral neuropathy, Sensory neuron disease, vasculitis-related neuropathy, Axonal peripheral neuropathy, Small fiber neuropathy, Infectious peripheral neuropathy, immune-related peripheral neuropathy, Ischemic peripheral neuropathy, Traumatic peripheral neuropathy, other endocrine and Polyneuropathy caused by metabolic diseases etc. |

# 2 Supplementary Figure


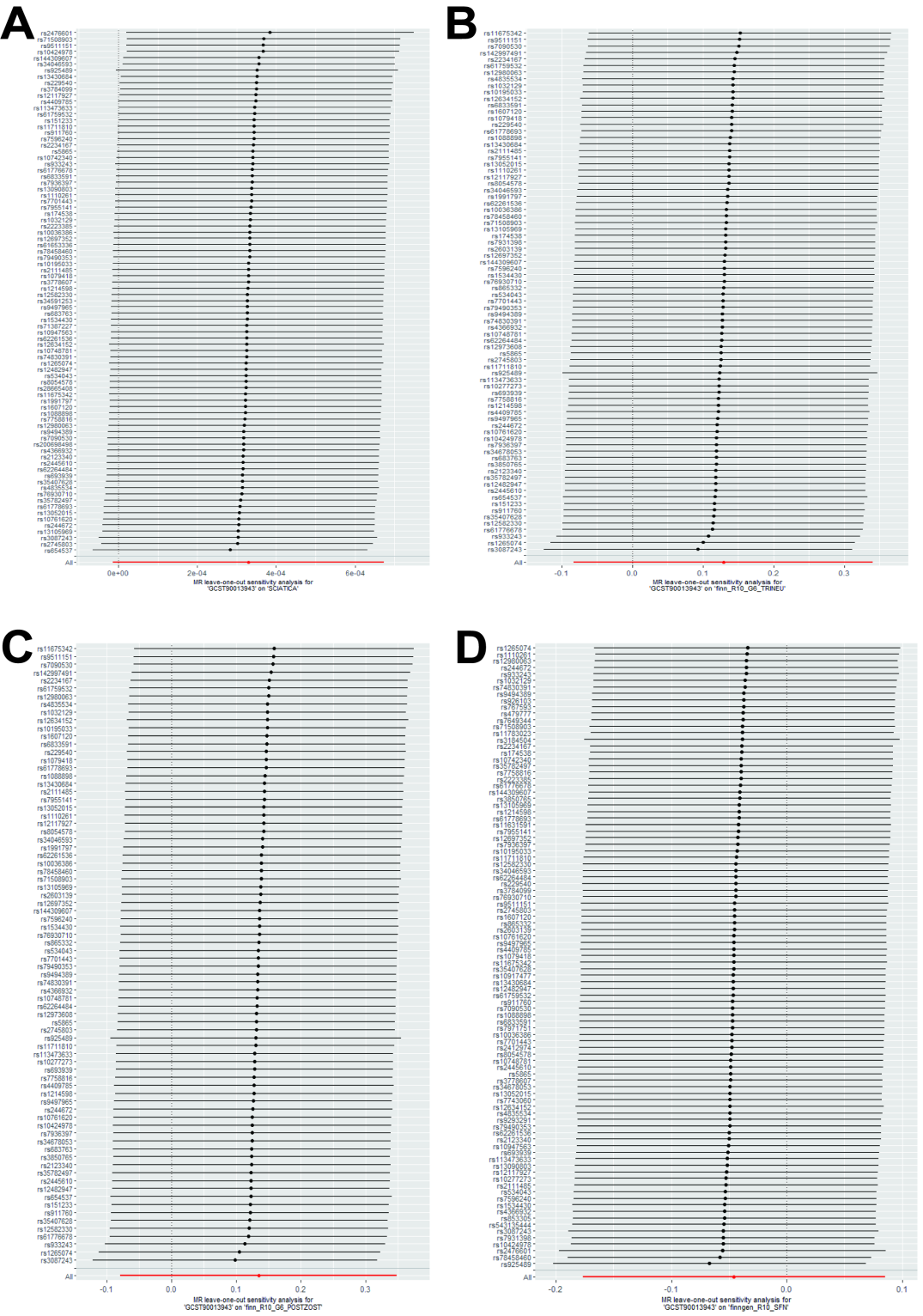


**Fig. S1 plots of leave-one-out analyses. (A)** hypothyroidism on sciatica with lumbago; **(B)** hypothyroidism on trigeminal neuralgia; **(C)** hypothyroidism on postzoster neuralgia **(D)** hypothyroidism on small fibre neuropathy.


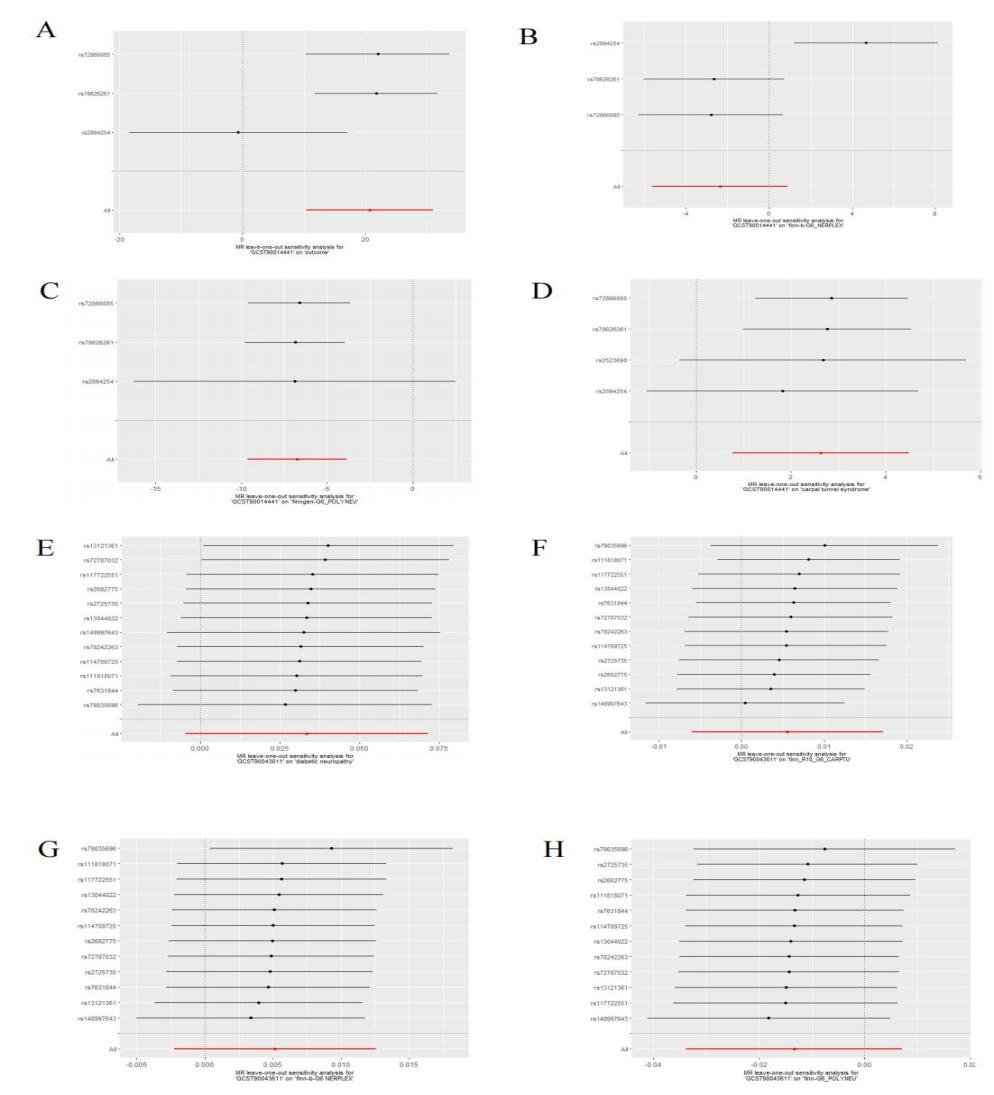


**Fig. S2** **plots of leave-one-out analyses. (A)** autoimmune thyroid disease on diabetic neuropathy; **(B)** autoimmune thyroid disease on nerve root/plexus disorder; **(C)** autoimmune thyroid disease on carpal tunnel syndrome **(D)** autoimmune thyroid disease on polyneuropathies. **(E)** benign neoplasm of pituitary gland and craniopharyngeal duct on diabetic neuropathy; **(F)** benign neoplasm of pituitary gland and craniopharyngeal duct on nerve root/plexus disorder; **(G)** benign neoplasm of pituitary gland and craniopharyngeal duct on carpal tunnel syndrome; **(H)** benign neoplasm of pituitary gland and craniopharyngeal duct on polyneuropathies.


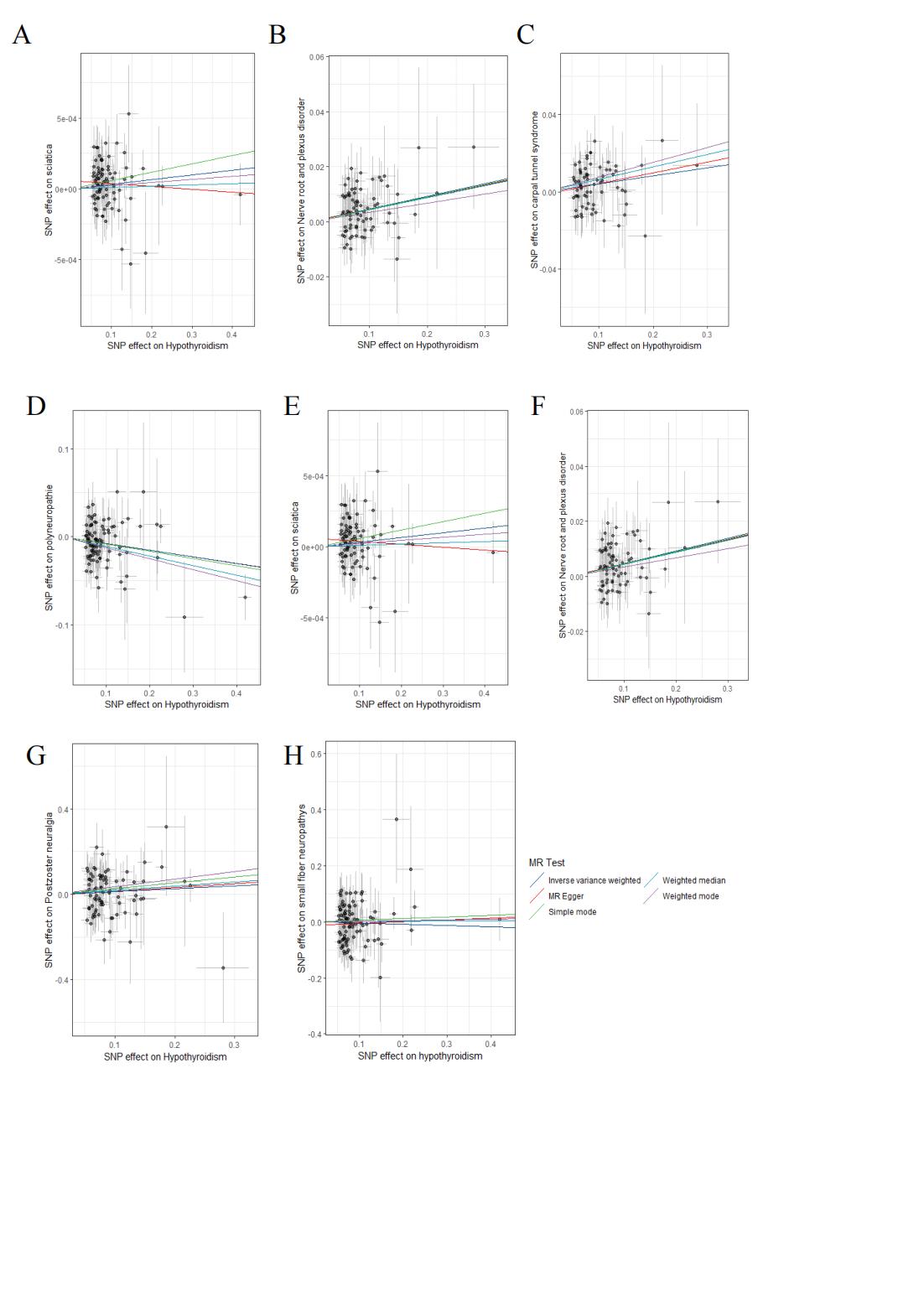


**Fig. S3 Scatter plots.** The X-axis represents the SNP effect on exposure, and the Y-axis represents the SNP effect on outcome. The slope > 0 indicates that the exposure factor will deepen the impact of the outcome factor.**(A)** hypothyroidism on diabetic neuropathy; **(B)** hypothyroidism on nerve root/plexus disorder; **(C)** hypothyroidism a on carpal tunnel syndrome; **(D)** hypothyroidism on polyneuropathies; **(E)** hypothyroidism on sciatica; (F) hypothyroidism on prosopalgia; **(G)** hypothyroidism on postherpetic neuralgia; **(H)** hypothyroidism on small fiber neuropathy.

**
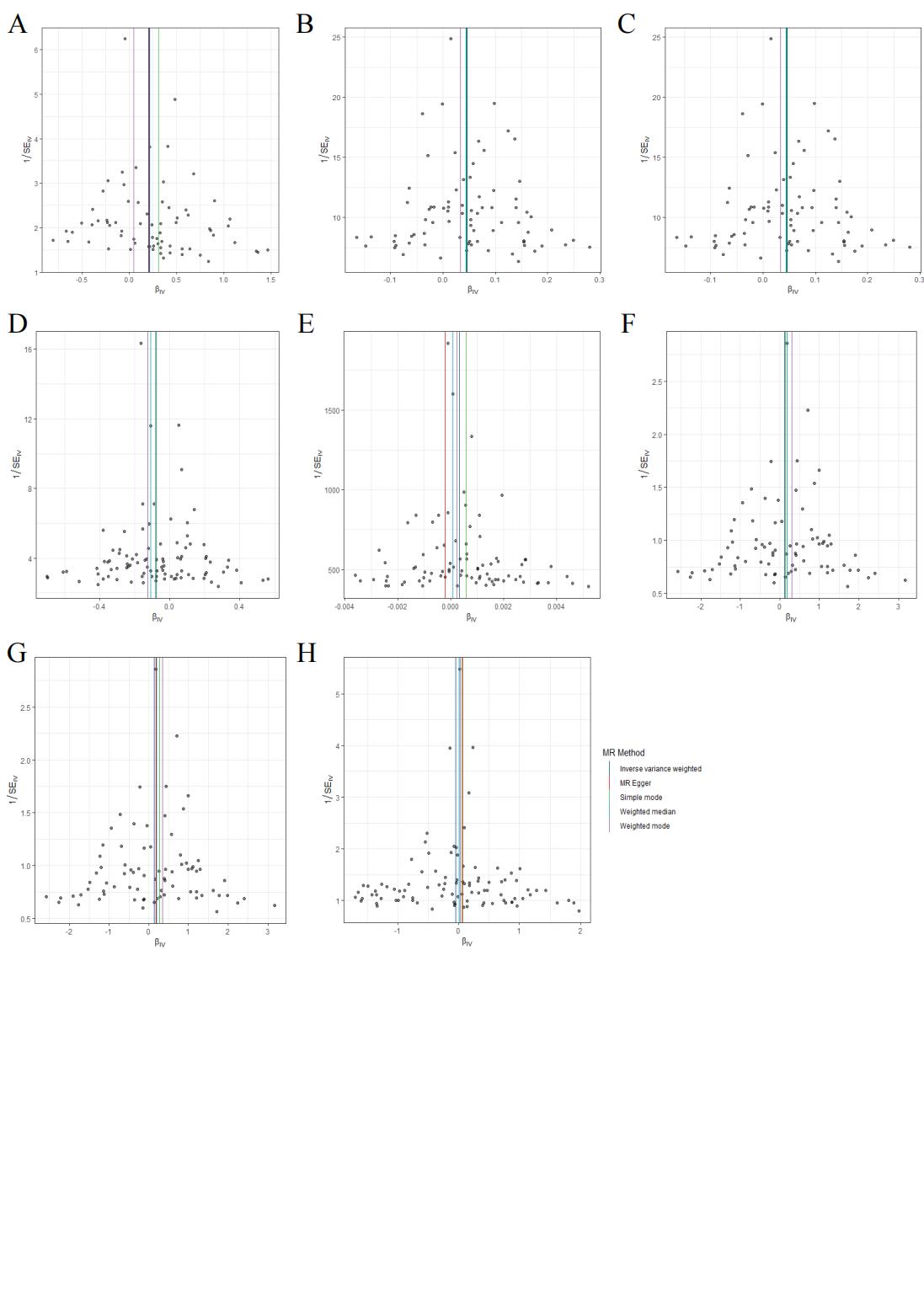
**

**Fig. S4 Funnel plots. (A)** hypothyroidism on diabetic neuropathy; **(B)** hypothyroidism on nerve root/plexus disorder; **(C)**  hypothyroidism on carpal tunnel syndrome; **(D)** hypothyroidism on polyneuropathies; **(E)** hypothyroidism on sciatica; (F) hypothyroidism on prosopalgia; **(G)** hypothyroidism on postherpetic neuralgia; **(H)** hypothyroidism on small fiber neuropathy.
