# Supplementary material for: Causal relationship between hypothyroidism and peripheral neuropathy: a Mendelian randomization study of European ancestry
Source: Front Endocrinol (Lausanne). 2024 Nov 27;15:1436823. doi: 10.3389/fendo.2024.1436823 (PMC11631618; doi:10.3389/fendo.2024.1436823)
Supplement: Supplementary file 3 [file DataSheet3.docx]

**STROBE-MR checklist of recommended items to address in reports of Mendelian randomization studies**^1^ ^2^

| **Item No.** | **Section** | **Checklist item** | **Relevant text from manuscript** |
| --- | --- | --- | --- |
| 1 | **TITLE and ABSTRACT** | Indicate Mendelian randomization (MR) as the study’s design in the title and/or the abstract if that is a main purpose of the study | Causal Relationship Between Hypothyroidism and Peripheral Neuropathy: A Mendelian Randomization Study of European Ancestry  Background: Metabolic disorders are significant risk factors for peripheral neuropathy (PN) diseases.....Conclusions: This study provides genetic evidence supporting potential causal links between hypothyroidism and various peripheral neuropathy diseases. |
|  | **INTRODUCTION** |  |  |
| 2 | **Background** | Explain the scientific background and rationale for the reported study. What is the exposure? Is a potential causal relationship between exposure and outcome plausible? Justify why MR is a helpful method to address the study question | Metabolic disorders are significant risk factors for peripheral neuropathy (PN) diseases. However, current clinical observational studies cannot fully determine the causal relationships between hypothyroidism (HT) and PN diseases. |
| 3 | **Objectives** | State specific objectives clearly, including pre-specified causal hypotheses (if any). State that MR is a method that, under specific assumptions, intends to estimate causal effects | In this study, we conducted two-sample MR analyses to explore the direct causal relationship between hypothyroidism and PN diseases. |
|  | **METHODS** |  |  |
| 4 | **Study design and data sources** | Present key elements of the study design early in the article. Consider including a table listing sources of data for all phases of the study. For each data source contributing to the analysis, describe the following: |  |
|  | a) | Setting: Describe the study design and the underlying population, if possible. Describe the setting, locations, and relevant dates, including periods of recruitment, exposure, follow-up, and data collection, when available. | Table 1 |
|  | b) | Participants: Give the eligibility criteria, and the sources and methods of selection of participants. Report the sample size, and whether any power or sample size calculations were carried out prior to the main analysis | Supplementary Table 1  2.2 Data sources |
|  | c) | Describe measurement, quality control and selection of genetic variants | 2.3 Genetic instrument selection |
|  | d) | For each exposure, outcome, and other relevant variables, describe methods of assessment and diagnostic criteria for diseases |  |
|  | e) | Provide details of ethics committee approval and participant informed consent, if relevant |  |
| 5 | **Assumptions** | Explicitly state the three core IV assumptions for the main analysis (relevance, independence and exclusion restriction) as well assumptions for any additional or sensitivity analysis | The MR analysis was grounded in three fundamental assumptions: 1) the chosen single nucleotide polymorphisms (SNPs) must show a statistically significant correlation with the exposure; 2) the SNPs should be independent of any confounders related to both the exposure and the outcome; 3) the SNPs should influence the outcome solely through the exposure and not through other direct or indirect pathways. |
| 6 | **Statistical methods: main analysis** | Describe statistical methods and statistics used | 2.4 Statistical analysis |
|  | a) | Describe how quantitative variables were handled in the analyses (i.e., scale, units, model) |  |
|  | b) | Describe how genetic variants were handled in the analyses and, if applicable, how their weights were selected |  |
|  | c) | Describe the MR estimator (e.g. two-stage least squares, Wald ratio) and related statistics. Detail the included covariates and, in case of two-sample MR, whether the same covariate set was used for adjustment in the two samples |  |
|  | d) | Explain how missing data were addressed |  |
|  | e) | If applicable, indicate how multiple testing was addressed |  |
| 7 | **Assessment of assumptions** | Describe any methods or prior knowledge used to assess the assumptions or justify their validity |  |
| 8 | **Sensitivity analyses and additional analyses** | Describe any sensitivity analyses or additional analyses performed (e.g. comparison of effect estimates from different approaches, independent replication, bias analytic techniques, validation of instruments, simulations) | For sensitivity analysis, we employed MR-Egger regression method and the leave-one-out approach. The P-value from the MR-Egger intercept test was used to evaluate potential horizontal pleiotropy in the IVs (28). Additionally, Cochrane’s Q statistic was utilized to assess the consistency of SNP estimates across each MR association. We also conducted a weighted median test to verify the directionality of each significant univariate MR result. |
| 9 | **Software and pre-registration** |  |  |
|  | a) | Name statistical software and package(s), including version and settings used | All analyses were conducted using the TwoSample MR (29) and RadialMR (26) packages in R software (version 4.3.1). |
|  | b) | State whether the study protocol and details were pre-registered (as well as when and where) |  |
|  | **RESULTS** |  |  |
| 10 | **Descriptive data** |  | 3.1 Instrumental variables selection |
|  | a) | Report the numbers of individuals at each stage of included studies and reasons for exclusion. Consider use of a flow diagram |  |
|  | b) | Report summary statistics for phenotypic exposure(s), outcome(s), and other relevant variables (e.g. means, SDs, proportions) |  |
|  | c) | If the data sources include meta-analyses of previous studies, provide the assessments of heterogeneity across these studies |  |
|  | d) | For two-sample MR:  i.  Provide justification of the similarity of the genetic variant-exposure associations between the exposure and outcome samples  ii.  Provide information on the number of individuals who overlap between the exposure and outcome studies |  |
| 11 | **Main results** |  | Figure 2  3.2 Univariable MR for the causal relationship between HT and PN diseases |
|  | a) | Report the associations between genetic variant and exposure, and between genetic variant and outcome, preferably on an interpretable scale | Table 2 |
|  | b) | Report MR estimates of the relationship between exposure and outcome, and the measures of uncertainty from the MR analysis, on an interpretable scale, such as odds ratio or relative risk per SD difference |  |
|  | c) | If relevant, consider translating estimates of relative risk into absolute risk for a meaningful time period |  |
|  | d) | Consider plots to visualize results (e.g. forest plot, scatterplot of associations between genetic variants and outcome versus between genetic variants and exposure) | Figure 2,3,4,5 |
| 12 | **Assessment of assumptions** |  |  |
|  | a) | Report the assessment of the validity of the assumptions | Table 3 |
|  | b) | Report any additional statistics (e.g., assessments of heterogeneity across genetic variants, such as *I^2^*, Q statistic or E-value) |  |
| 13 | **Sensitivity analyses and additional analyses** |  |  |
|  | a) | Report any sensitivity analyses to assess the robustness of the main results to violations of the assumptions | Additionally, a series of sensitivity analyses assessed the robustness of these findings. Heterogeneity was detected in the analysis between autoimmune thyroid disease (AITD) and nerve root/plexus disorder, and CTS, according to Cochran's Q statistic, but no horizontal pleiotropy was detected as per the MR-Egger intercept test (Table 4). |
|  | b) | Report results from other sensitivity analyses or additional analyses |  |
|  | c) | Report any assessment of direction of causal relationship (e.g., bidirectional MR) | We performed the Steiger directionality test after the univariable MR analysis, confirming that all results were in the correct positive direction (Table 3). |
|  | d) | When relevant, report and compare with estimates from non-MR analyses |  |
|  | e) | Consider additional plots to visualize results (e.g., leave-one-out analyses) | The funnel plots indicated a symmetric distribution (Supplementary Figure 3), and the "leave-one-out" method showed that removing any SNP had minimal impact on the overall error line (Figure 5, Supplementary Figure 1). |
|  | **DISCUSSION** |  |  |
| 14 | **Key results** | Summarize key results with reference to study objectives | In our MR study of hypothyroidism and eight PN diseases, three MR analyses were conducted by using European ancestry GWAS data to detect the relationship between hypothyroidism and PN diseases risk. we found that hypothyroidism is a risk factor of diabetic neuropathy, nerve root/plexus disorder, and carpal tunnel syndrome, while acted as a oppose causality for polyneuropathy. Further analysis showed that autoimmune thyroid disease is a risk factor of carpal tunnel syndrome. However, we observed no statistically significant association between benign neoplasm of the pituitary gland and craniopharyngeal duct and PN diseases. |
| 15 | **Limitations** | Discuss limitations of the study, taking into account the validity of the IV assumptions, other sources of potential bias, and imprecision. Discuss both direction and magnitude of any potential bias and any efforts to address them | However, the study has several limitations. Firstly, the GWAS data were solely from individuals of European descent (U.K. and Finland), potentially limiting the generalizability of our findings. Secondly... Lastly, we excluded single nucleotide polymorphisms (SNPs) associated with known confounders, but further studies are required to address other unknown confounders. |
| 16 | **Interpretation** |  |  |
|  | a) | Meaning: Give a cautious overall interpretation of results in the context of their limitations and in comparison with other studies | As for diabetic neuropathy, carpal tunnel syndrome and polyneuropathies, previous clinical observations were consistent with our findings. |
|  | b) | Mechanism: Discuss underlying biological mechanisms that could drive a potential causal relationship between the investigated exposure and the outcome, and whether the gene-environment equivalence assumption is reasonable. Use causal language carefully, clarifying that IV estimates may provide causal effects only under certain assumptions | The underlying mechanisms through which hypothyroidism contributes to neuropathy remain insufficiently explored. Recent research has identified that impaired transport of thyroid hormones to the brain results in cerebral hypothyroidism, which subsequently induces astrogliosis and disrupts both the metabolic function and mitochondrial respiration of astrocytes (31). One proposed mechanism for nerve damage associated with hypothyroidism involves fluid retention, which may lead to tissue swelling and the subsequent compression of peripheral nerves (32). |
|  | c) | Clinical relevance: Discuss whether the results have clinical or public policy relevance, and to what extent they inform effect sizes of possible interventions | Clinical observational studies suggest an association between hypothyroidism and peripheral neuropathy (8, 9, 11-13).  A case study of a 30-year-old male with severe hypothyroidism due to chronic autoimmune thyroiditis revealed severe sensorimotor polyneuropathy with both axonal and demyelinating features through nerve conduction studies (10). |
| 17 | **Generalizability** | Discuss the generalizability of the study results (a) to other populations, (b) across other exposure periods/timings, and (c) across other levels of exposure | An 11-year study in a South Korean population found that hypothyroidism was not a risk factor for CTS (45). |
|  | **OTHER INFORMATION** |  |  |
| 18 | **Funding** | Describe sources of funding and the role of funders in the present study and, if applicable, sources of funding for the databases and original study or studies on which the present study is based | This study is supported by the National Natural Science Foundation of China (82074163), the Shanghai Clinical Research Center of Acupuncture (20MC1920500), key construction discipline of Natioanl Administration of Traditional Chinese Medicine (ZYYZDXK-2023068), and Clinical Key Specialty Construction Foundation of Shanghai (shslczdzk04701). The funding sources have no role in the design of the study, and will not have any involvement in its execution, data analysis, interpretation, or decision to submit the results. |
| 19 | **Data and data sharing** | Provide the data used to perform all analyses or report where and how the data can be accessed, and reference these sources in the article. Provide the statistical code needed to reproduce the results in the article, or report whether the code is publicly accessible and if so, where | GWAS Catalog (https://www.ebi.ac.uk/gwas/) and IEU open GWAS catalog (https://gwas.mrcieu.ac.uk/). FinnGen (https://www.finngen.fi/en/access_results) |
| 20 | **Conflicts of Interest** | All authors should declare all potential conflicts of interest | All financial, commercial or other relationships that might be perceived by the academic community as representing a potential conflict of interest must be disclosed. If no such relationship exists, authors will be asked to confirm the following statement:  The authors declare that the research was conducted in the absence of any commercial or financial relationships that could be construed as a potential conflict of interest. |

This checklist is copyrighted by the Equator Network under the Creative Commons Attribution 3.0 Unported (CC BY 3.0) license.

1. Skrivankova VW, Richmond RC, Woolf BAR, Yarmolinsky J, Davies NM, Swanson SA, et al. Strengthening the Reporting of Observational Studies in Epidemiology using Mendelian Randomization (STROBE-MR) Statement. JAMA. 2021;under review.

2. Skrivankova VW, Richmond RC, Woolf BAR, Davies NM, Swanson SA, VanderWeele TJ, et al. Strengthening the Reporting of Observational Studies in Epidemiology using Mendelian Randomisation (STROBE-MR): Explanation and Elaboration. BMJ. 2021;375:n2233.
